# Increase and Change in the Pattern of Antibiotic Use in Serbia (2010–2019)

**DOI:** 10.3390/antibiotics10040397

**Published:** 2021-04-07

**Authors:** Ana Tomas, Nebojša Pavlović, Nebojša Stilinović, Olga Horvat, Milica Paut-Kusturica, Tihomir Dugandžija, Zdenko Tomić, Ana Sabo

**Affiliations:** 1 Department of Pharmacology, Toxicology and Clinical Pharmacology, Faculty of Medicine, University of Novi Sad, Hajduk Veljkova 3, 21000 Novi Sad, Serbia; nebojsa.stilinovic@mf.uns.ac.rs (N.S.); olga.horvat@mf.uns.ac.rs (O.H.); milica.paut-kusturica@mf.uns.ac.rs (M.P.-K.); zdenko.tomic@mf.uns.ac.rs (Z.T.); ana.sabo@mf.uns.ac.rs (A.S.); 2 Department of Pharmacy, Faculty of Medicine, University of Novi Sad, Hajduk Veljkova 3, 21000 Novi Sad, Serbia; nebojsa.pavlovic@mf.uns.ac.rs; 3 Department of Epidemiology, Faculty of Medicine, University of Novi Sad, Hajduk Veljkova 3, 21000 Novi Sad, Serbia; tihomir.dugandzija@mf.uns.ac.rs; 4 Oncology Institute of Vojvodina, Put doktora Goldmana 4, 21204 Sremska Kamenica, Serbia

**Keywords:** antibiotics, Serbia, ATC/DDD, drug utilization research, AWaRe classification

## Abstract

The aim of this study was to determine and describe trends in antibiotics utilization in Serbia over a ten-year period. Data were retrieved from publicly available annual reports (2010–2019). The results were expressed as Defined Daily Dose (DDD) per 1000 inhabitants per day (DID). All calculations were performed using the DDD values for the 2020 Anatomical Therapeutic Chemical/Defined Daily Dose (ATC/DDD) version for each year of the study, to account for the DDD changes during the study period. Antibiotics were classified using the WHO Access, Watch, Reserve (AWaRe) classification. Total utilization of antibacterials for systemic use increased from 17.25 DID in 2010 to 28.65 DID in 2019. A statistically significant increasing trend in the use of the Watch category antibiotics was observed. A tendency towards use of broad-spectrum antibiotics, apparent by a statistically significant increase in the rate of utilization of broad-spectrum macrolides, quinolones and third-generation cephalosporins vs. narrow-spectrum ones, as well as a significant increasing trend in the use of quinolones was identified. Total antibiotic utilization was found to be well above the European average. Several specific problem areas were identified, which requires further efforts to improve antibiotic prescribing. The present study provides the information needed to facilitate antibiotic stewardship in Serbia further and proposes specific interventions to optimize antibiotic use in Serbia.

## 1. Introduction

Antimicrobial resistance (AMR) is one of the biggest challenges of the 21st century, identified by the World Health Organization (WHO) as one of the top 10 global public health threats humanity is facing [1]. A global action plan (GAP) on antimicrobial resistance was endorsed at the World Health Assembly in May 2015 with a number of strategic objectives [2]. These include improved awareness and understanding of antimicrobial resistance, strengthening surveillance and research, reducing the incidence of infections, optimizing the use of antimicrobials and ensuring sustainable investment in countering AMR. This is achieved through controlling infection and optimizing drug use. These activities include providing adequate water supply, sanitation and hygiene, infection prevention and control, immunization, laboratory services, workforce education and antibiotic supply chain management [3]. A core strategy for controlling resistance is to coordinate efforts through a national action plan on AMR. Since 2015, more than 115 countries have developed national action plans to tackle AMR, aligned with the GAP, establishing AMR coordination committees to deliver them [3].

The use of antibiotics is one of the most important modifiable risk factors for the development of AMR. In Serbia, significant steps have been made over the past decade to reduce the irrational use of antibiotics. These include changes in legislation and a ban on over-the-counter antibiotic sales and better control of antibiotic dispensing [4,5]. In the last trimester of 2011, more stringent control of issuing of antibiotics was implemented to limit the over-the-counter sales of antibiotics in pharmacies [6]. A campaign for the rational consumption of antibiotics primarily aimed at the health professionals was launched and two national-level guidelines for rational antibiotic use were published, the most recent one in 2018 [7]. Following all this effort, total antibiotic consumption in Serbia for systemic use reduced by more than 30% between 2015 and 2017. In 2019, a national antibiotic resistance control programme for the period 2019–2021 was introduced in the Republic of Serbia. It lays down the objectives, activities and procedures to be carried out to stop the spread of antibiotic resistance, including encouraging research into aspects of AMR at the national and international level [8].

Monitoring antibiotic consumption in both the in- and out-patient settings is of utmost importance as it provides the basis for the implementation and evaluation of antibiotic stewardship interventions. The Serbian Agency for Drugs and Medical Devices publishes yearly reports containing aggregated data on antibiotic consumption based on wholesale data [9], including consumption in in- and out-patient settings. Reports are based on the WHO ATC/DDD (Anatomical Therapeutic Chemical/Defined Daily Dose) methodology [10]. However, the DDDs used for calculations are subjected to change, and the already published, available reports do not get retroactively updated with regard to changes in DDDs. Because of this, direct determination and comparison of temporal trends in antibiotic use based solely on public reports are difficult. When publishing longitudinal drug utilization studies, drug utilization data should be calculated using the ATC/DDD index version of the last year of the time window for each year [11]. 

More specifically, in 2019, the ATC Index with DDDs included substantial alterations in DDDs for eight antibacterials. The impact of the changes in DDDs on the patterns of antibiotic consumption differs, but in general, application of the new DDDs has the largest impact on consumption of penicillins, and comparing the trends in antibiotic use with reports published prior to this change in DDDs without accounting for this alteration leads to misinterpretation. A paper that reports on the impact of a change in amoxicillin DDD on the overall consumption of antimicrobials found that changes in total DID (Defined Daily Dose/1000 inhabitants/day) before and after the 2019 DDD adjustment ranged from 5.1% in Norway to as high as 19.2% in Spain [12]. Logically, in countries where amoxicillin is more commonly used, the total utilization changed more significantly. In Serbia, amoxicillin (alone or in combination with beta-lactamase inhibitors), as well as other penicillins, are the most commonly used antibiotics based on consumption expressed in DID [4]. 

Taking into account the above, this study aimed to determine and describe the trends and patterns in antibiotic utilization in Serbia, while accounting for the recent DDD adjustments. Secondly, this paper aimed to identify potential problem areas in antibiotic use by employing the WHO Access, Watch, Reserve (AWaRe) classification of 2019. 

## 2. Results

**Figure 1:**
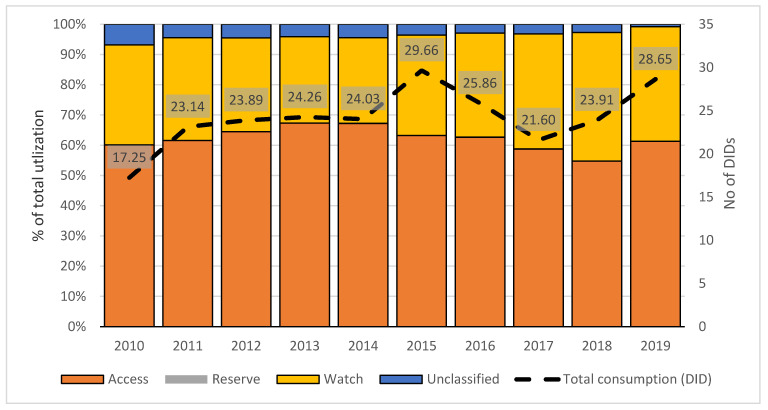
Trends in antibiotic utilization over a 10-year period in Serbia according to the WHO Access, Watch, Reserve (AWaRe) classification. Bars show the share of the different Access, Watch and Reserve categories in overall antibiotic utilization, while the line graph displays total antibiotic utilization in Defined Daily Dose/1000 inhabitants/day (DID) between 2010 and 2019.

The overall statistical trends for antibiotic utilization and shares of different categories according to the WHO AWaRe classification over a 10-year period in Serbia are shown in Figure 1. Total utilization of antibacterials for systemic use (J01 group) increased from 17.25 DID in 2010 to 29.66 DID in 2015, which was followed by a decrease in utilization to 21.60 in 2017 and an increase to 28.65 DID in 2019. An overall increasing trend in total antibiotic utilization between 2010 and 2019 was observed, but it was not significant (β = 0.565, *p* = 0.089). A statistically significant increasing trend in the use of Watch category antibiotics (33.08% in 2010 to 37.93% in 2019; β = 0.666, *p* = 0.037) was observed. An overall increasing trend was observed, but without statistical significance for the Reserve (0.006% in 2010 to 0.02% in 2019; β = 0.601, *p* = 0.066) and Access (60.14% in 2010 to 61.27% in 2019; β = 0.667, *p* = 0.243) categories of antibiotics.

The overall statistical trends for utilization of penicillins (J01C_DID), cephalosporins (J01D_DID), macrolides, lincosamides and streptogramins (J01F_DID) and quinolones (J01M_DID) expressed in DID are shown in Table 1. A statistically significant increasing trend in the use of quinolone antibiotics (J01M, 2.10 DID in 2010 to 3.23 in 2019; β = 0.667, *p* = 0.035) was observed. An overall increasing trend in utilization of penicillins (J01C_DID), cephalosporins (J01D_DID) and macrolides, lincosamides and streptogramins (J01F_DID) between 2010 and 2019 was observed, but it was not statistically significant.

Changes in the values of the quality indicators describing the relative use of beta-lactamase-sensitive penicillins (J01CE), penicillins, including beta-lactamase inhibitor (J01CR), third- and fourth-generation cephalosporins (J01DD + J01DE) and fluoroquinolones (J01MA), as well as the ratio of utilization of broad-spectrum to narrow-spectrum penicillins, cephalosporins and macrolides (J01_B/N) over a 10-year period in Serbia are shown in Table 2. There was a decrease in the share of narrow spectrum penicillins in total antibiotic utilization (J01CE_%, 1.08% in 2010 to 0.26% in 2019). There was a statistically significant increasing trend in the share of utilization of penicillins, including beta-lactamase inhibitor (J01CR_%, 6.57% in 2010 to 21.41% in 2019; β = 0.772, *p* = 0.009), third- and fourth-generation cephalosporins (J01DD + J01DE_%, 4.01% in 2010 to 5.53% in 2019; β = 0.664, *p* = 0.036) and fluoroquinolones (J01MA_%, 6.64% in 2010 to 11.16% in 2019; β = 0.786, *p* = 0.007) in total antibiotic utilization. The ratio of utilization of broad-spectrum to narrow-spectrum penicillins, cephalosporins and macrolides (J01_B/N) increased significantly (β = 0.692, *p* = 0.027), from 1.42 in 2010 to 4.16 in 2019. This finding is the consequence of utilization of narrow-spectrum penicillins, cephalosporins and macrolides remaining stable between 2010 and 2019 (3.29 vs. 3.26 DID), while there was a statistically significant increase in the utilization of broad-spectrum agents of these classes (4.69 DID in 2010 to 13.56 DID in 2019, β = 0.767, *p* = 0.010).

When trends in consumption of all individual agents were assessed (Table 3), a significant change was found for the number of antibiotics. There was a significant decrease in the use of pipemidic acid (−86.26% relative decrease, *p* = 0.001), cefaclor (−83.66%, *p* = 0.008), tetracycline (−82.42%, *p* = 0.002), roxithromycin (−76.71%, *p* = 0.037) and gentamycin (−21.58%, *p* = 0.026). A significant increasing trend was found in the utilization of 15 individual antibiotics, out of which 4 belonged to the Access group (cefadroxil 61.63% relative increase, *p* = 0.064; cefazolin 331.50%, *p* = 0.045; amoxicillin, clavulanic acid 442.00%, *p* = 0.014; clindamycin 1421.00%, *p* = 0.001). A significant increasing trend was found for the 9 Watch category antibiotics (clarithromycin 74.79% relative increase, *p* = 0.004; ceftazidime 92.65%, *p* = 0.047; cefixime 114.55%, *p* = 0.009; tobramycin 132.80%, *p* = 0.012; ciprofloxacin 160.56%, *p* = 0.028; levofloxacin *p* < 0.001; vancomycin 70.00%, *p* = 0.02; fosfomycin 373.20%, *p* < 0.001), and for 2 antimicrobials belonging to the Reserve category (tigecycline 510.00%, *p* = 0.018 and linezolid 1318.65%, *p* < 0.001).

Considerable variation, both in the pattern and amount of antibiotics used at the beginning of the study (2010), year at which the total utilization peaked (2015) and the last available data (2019), was found when the drug utilization 90% (DU90%) profiles were determined (Table 4). In 2010 and 2015, amoxicillin was the most commonly used antibiotic in Serbia, followed by cephalexin. In 2019, co-amoxiclav ranked first, followed by amoxicillin. A total of 43–48 substances (according to the generic names) of systemic antibiotics were used; moreover, there were 12–15 different substances within the DU90% segment. The number of antibiotics in the DU90% segment accounted for 25–34.88% of the total number of different antibiotics used. Within the DU90% segment, the number of beta-lactam antibacterials (ATC codes J01C and J01D) varied from 5 to 6 in each of the years. Among the macrolides, azithromycin and clarithromycin ranked up from 5th and 8th in 2010 to 3rd and 5th place in 2019.

The share of the Watch category antibiotics in the total number of different agents was 53.30% in 2010 and 53.84% in 2019, and they contributed to 28.87% and 33.86% of the total utilization in 2010 and 2019, respectively. From the Access group, there were between 5 and 6 antibiotics within the DU90%, accounting for 56.67% and 56.33% of the total utilization in 2010 and 2019, respectively. There were no Reserve antibiotics within the DU90% in any of the years included in the study.

## 3. Discussion

Reporting on antibiotic utilization is an essential element of surveillance, providing important information in the global fight against AMR [13]. To the best of our knowledge, this is the first study applying the 2019 WHO AWaRe classification in temporal analysis of antibiotic consumption in Serbia. This updated version covers the non-essential antibiotics, which minimizes the shares of unclassified antibiotics in our study, commonly found in previous studies. The study also reflects on several quality indicators for the use of antibiotics in Serbia, covering a 10-year period, which was also not previously reported. During the analysis, the changes in DDDs during the study period were addressed, allowing the accurate representation of temporal evolution of antibiotic utilization. 

According to the results of this longitudinal national study on the utilization of antibiotics in a ten-year period in Serbia, the overall increasing trend in total consumption of antibacterials for systemic use between 2010 and 2019 was observed. Even though the antibiotic utilization decreased from 29.66 DID to 21.60 between 2015 and 2017, this was followed by an increase to 28.65 DID in 2019. In the study period, there were no substantial changes in the burden of communicable diseases that might have driven the increase in antibiotic utilization. According to the “Report on Infectious Diseases in Republic of Serbia for 2018”, published by the Institute of Public Health of Serbia, the infectious diseases incidence rates show a decreasing trend in the period between 2010 and 2017 [14]. Similarly, based on The Global Burden of Disease Study data, the prevalence rate for communicable, maternal, neonatal and nutritional diseases in Serbia declined during the 2010–2019 period (from 41,882 in 2010 to 38,054 in 2019). Specifically, for the communicable diseases with the highest prevalence, including upper respiratory infections, chlamydial infections and otitis media, the prevalence rate decreased or remained stable during the whole study period and more specifically since 2017, when the increase in antibiotic utilization occurred in Serbia [15]. With total antibiotic utilization of 29.66 DID in 2019, Serbia ranks third in Europe, with only Cyprus (30.1 DID) and Greece (34.1 DID) reporting higher consumption, according to the European Centre for Disease Prevention and Control’s latest report [16]. In 2019, the average total consumption (community and hospital sector) of antibacterials for systemic use in 30 different countries across Europe was 19.4 DID (country range: 9.5–34.1). During the period 2010–2019, a statistically significant decrease was observed for the European countries overall [16]. However, in a multinational study conducted across 76 countries, a sharp rise in antibiotic consumption, based on drug sales data, was determined in a period 2000–2015, especially in low- and middle-income countries (+114% vs. 65% globally) [17]. 

A recent study demonstrated that the main reasons for the increasing trend in total consumption of antibiotics may lie in inappropriate prescription practices in primary care across low- and middle-income countries [18]. To help optimize, monitor and report antibiotic use, the WHO developed the AWaRe classification system for antibacterials, which includes the Access, Watch, Reserve categories, in the context of a comprehensive review of the optimal antibiotic choices for many common infectious syndromes in adults and children. This framework can be used for national antimicrobial stewardship programs, with the main aims of improving access and clinical outcomes, reducing AMR and preserving the effectiveness of last-line antibiotics at the same time [19]. Measuring the absolute or relative use of antibiotics in each of the AWaRe categories may indicate the overall quality of antibiotic use in a country [20]. The indicator of at least 60% of antibiotic consumption being from medicines in the Access category was included to monitor access to essential medicines and progress towards Universal Health Coverage (UHC) by the WHO [21]. An increasing trend in the use of all AWaRe categories of antibiotics was observed in the 2010–2019 period in Serbia. During this ten-year period, the consumption of the Access group was usually slightly above 60%, although in 2017 and 2018 it was 58.7% and 54.7%, respectively. Reserve group antibiotics accounted for 0.01% of the total consumption, and there were no Reserve antibiotics within the DU90% in any of the years included in the study. Based on the WHO Report on Surveillance of Antibiotic Consumption 2016–2018, the Access group antibiotics accounted for >50% of the total consumption in 34 out of 45 countries (median proportional consumption 56% (IQR: 49–64%)), with the values ranging from 38% in Albania to 78% in Iceland [13]. Reserve group antibiotics were only rarely used in most countries and areas, with a median proportional use of 0.2% (IQR: 0.1–0.5%) [13].

In the present study, a statistically significant increasing trend was observed in the use of Watch category antibiotics between 2010 and 2019 (*p* = 0.037), with a maximal value in 2018 (42.5%). According to the abovementioned WHO report, the median proportion of Watch group antibiotics was 29% (IQR: 25–36%), with values ranging from less than 20% in the Nordic countries (Denmark, Finland, Iceland, Norway and Sweden) to 52% in Georgia [13]. Similar to the results of our study, the share of Watch category antibiotics prescribed in Swiss primary care was 42% in the 2008–2020 period [22]. Community-level consumption of Watch category antibiotics was around 40% in Vietnam in the 2017–2018 period, and the supply from private pharmacies, non-prescription antibiotic sale and the use in children were identified as factors associated with a higher likelihood of Watch-group antibiotic use [23]. In Colombia, the share of Watch-group antibiotics was only 17% of all prescribed antibiotics in 2020, and living in a municipality increased the probability of receiving Watch- or Reserve-group antibiotics in comparison to living in capital cities [24]. On the other hand, very high proportions of Watch-group antibiotics were identified in Syria (66% in 2018–2019) [25] and Kazakhstan (61–68% in 2017–2019 period) [26], indicating the irrational use of antibiotics and the need for the improvement of current national policies in order to prevent the spread of AMR. 

In our study, the Watch-group antibiotics were mainly represented by macrolides (azithromycin, clarithromycin) but also fluoroquinolones (ciprofloxacin and levofloxacin) and second- and third-generation cephalosporins (cefixime, ceftriaxone, cefuroxime). The Watch-group antibiotics have a higher resistance potential when compared to the Access-group antibiotics [27], where azithromycin, with a proportion of around 10% of all antibiotics, stood out as the most used Watch-group antibiotic in Serbia in 2019. There are limited data about the antibiotic prescribing habits of physicians in Serbia, but a recent paper identified the over-prescription of antibiotics (mostly macrolides and beta-lactams) by physicians, as these agents were prescribed to the majority of patients with a diagnosis of acute bronchitis (78.5%), in contrast to the recommendations in the national guidelines [28]. However, penicillins (J01C) were the most frequently used group of antibiotics in Serbia, with a proportion of 32.9–39.3% in the 2010–2019 period, which is consistent with the results of studies conducted in European countries and the United States [16,29,30]. Amoxicillin was the most commonly used antibiotic, alone or in combination with clavulanic acid, and its use increased from 4.18 DID in 2010 to 10.89 DID in 2019, which represents 38% of all used antibacterials in Serbia in 2019. The utilization of all main ATC groups of antibiotics increased between 2010 and 2019 in Serbia; however, this upward trend was statistically significant only for quinolones (J01M). On the contrary, in Swiss primary care, fluoroquinolones and macrolides/lincosamides prescriptions significantly declined by 53% and 51% between 2008 and 2020. However, this decline might have been at the expense of increased and potentially inappropriate use of broad-spectrum penicillins [22].

Several findings clearly identify the area where there is the biggest room for improvement in antibiotic use in Serbia—over-use of broad-spectrum antibiotics. This finding is supported by the statistically significant increase in the use of Watch-category antibiotics, the statistically significant increase in quinolones use and the increase in the ratio of broad-spectrum penicillins, cephalosporins and macrolides compared to the narrow-spectrum agents of these classes. Furthermore, when the DU90% profile was determined, it was obvious that among the most commonly used antimicrobials, accounting for 90% of the total utilization, the share of the Watch-group antibiotics of the total number of different agents within DU90% was 53.84% (7/13), on the account of macrolides, quinolones and third-generation cephalosporins. An increasing trend in the utilization of fluoroquinolone was observed in Serbia (11.16% in 2019), which was also used at a high level compared to most other European countries. Based on this parameter, Serbia ranks 5th among all 33 countries with available data [31], with only Cyprus (19.3%), Bulgaria (14.5%), Hungary (14.5%) and Romania (13.00%) reporting a higher share of quinolones in their total antibiotic utilization. The ratio of broad-spectrum penicillins, cephalosporins and macrolides to the narrow-spectrum agents of these classes increased from 1.42 in 2010 to 4.16 in 2019, which is similar to what is reported for Czechia (5.2) and Ireland (4.29), much lower than in Greece, but higher than in Slovenia (3.33), the UK (1.93) and Nordic countries (<1) [31]. There are also concerns about the low level of use of beta-lactamase-sensitive penicillins in Serbia, compared to, for instance, Slovakia, Finland, Slovenia or Sweden [32]. According to the share of third- and fourth-generation cephalosporins (J01(DD + DE)) in total antibiotic utilization (5.53% in 2019), Serbia ranks 2nd, with only Italy reporting a higher share (10.10%). The results are comparable to those of Bulgaria (5.20%) and Slovakia (4.70%), but much higher than those reported in West European countries, but also countries with a similar healthcare system and historical and cultural background (Croatia, 2.90% and Slovenia, 0.60%). Furthermore, when individual agents were assessed, out of the Watch group, a significant increasing trend was found for macrolides (clarithromycin), third-generation cephalosporins (ceftazidime, cefixime) and quinolones (ciprofloxacin, levofloxacin), confirming the overall tendency to use these broad-spectrum agents more commonly than narrow-spectrum antibiotics. Broad-spectrum antibiotics, such as third-generation cephalosporins, and quinolones should be used with caution because of their high potential to cause the development of AMR and side-effects [20]. In Serbia, based on the Central Asian and Eastern European Surveillance of Antimicrobial Resistance (CAESAR) reports [33,34], between 2014 and 2019, resistance levels in *P. aeruginosa* and *E. coli* to quinolones increased from 47% to 59% and 30% to 35%, respectively. In 2019, 56% of all *P. aeruginosa* isolates in Serbia were multidrug resistant. For *K. pneumoniae*, high levels of resistance, including carbapenems, more than 70% to quinolones and more than 80% to third-generation cephalosporins, were present in 2019. High levels of resistance were also reported for penicillin and macrolides in *S. pneumoniae* [33]. There is a strong association between levels of antimicrobial use and AMR, and one of the main goals of the WHO global action plan on AMR is to optimize antibiotic use [35]. 

The Serbian government has put in significant effort to confine AMR through implementation of a series of policies and measures, including establishing a national program for the control of bacterial resistance to antibiotics, changes in legislation for antibiotic dispensing, public campaigns and national guidelines, yet the total utilization remains high above the European average. A tendency towards the use of broad-spectrum antibiotics, macrolides, quinolones and third-generation cephalosporins needs to be addressed. Exact factors driving these findings are difficult to completely grasp. Over 80% of antibiotics in Serbia are prescribed in the outpatient setting [4], and according to the literature, patient expectations, prescribing practices of medical colleagues, cultural factors, professional etiquette and uncertainty of the diagnosis coupled with patient expectations for antibiotics apply prescribing pressure on GPs [36]. There is a number of evidence-based interventions that can be tailored to the specific setting, such as implementation of individual feedback on antibiotic prescriptions to prescribers, which was found to cause improvements in guideline adherence, as was shown in a number of studies [37,38]. Other than the strategies of audit and feedback, educational outreach visits were found to be effective in improving the process of care, with a consistent effect on prescription improvement [39,40]. Strategies such as shared decision making, utilized to educate patients about when antibiotics are and are not needed, have been shown to reduce the prescribing of antibiotics for acute respiratory infections from 47 to 29% in comparison with standard management [41]. Efforts to improve antibiotic prescribing, relying on multi-sectorial collaboration at the local and national levels, should combine physician, patient and public education, taking into account local cultural context [42]. The first step is identifying stewardship targets. The current situation in Serbia, with an increasing trend in the use of Watch-group antibiotics, implies the need for encouraging the use of first-line antibiotics to treat common infections. Therefore, prescribers need to be motivated to include the AWaRe classification in the decision-making processes. Prior to this, the AWaRe classification should be rechecked and, if necessary, adapted to the Serbian setting, in relation to the recommendations in national guidelines and AMR rates [43]. Considering the high use of amoxicillin/clavulanic in the Serbian setting, this antibiotic might need to be reclassified to the Watch group, to avoid overuse and resistance increasing. However, for the AWaRe index to be embedded into national antibiotic stewardship policy, a joint effort of decision- and policy-makers is required. A number of successfully implemented policies around the globe confirm the impact that appropriate antibiotic stewardship can have on antibiotic use and AMR. For example, in Australia, the use of fluoroquinolones has long been restricted by guidelines favoring alternative options, and the limitation of the prescription subsidies for this antibiotic class to very specific indications. As a consequence, Australia is one of the lowest overall users of fluoroquinolones among high-income countries, and despite a relatively high overall antibiotic use in the community, the country’s rates of resistance to fluoroquinolones are low compared to many European countries [44]. In the UK, the introduction of financial incentives for local commissioners of healthcare to improve the quality of prescribing was associated with a significant reduction in both total and broad-spectrum antibiotic prescribing in primary care in England [45]. In Sweden, a stepwise development of the strategic programme against antibiotic resistance started with the formation of the Swedish strategic programme against antibiotic resistance, also known as Strama, in 1995. According to the >20-year-long Swedish experience, working closely with prescribers at the local level has been the key element for achieving long-term change [46]. Levels of antibiotic use and resistance in Sweden are now among the lowest among the European Union (EU) countries, both in the human and animal sectors. Successful antimicrobial stewardship models in European countries start with a long early adoption phase at the practice level, thereby followed by policy changes that eventually lead to large-scale adoption [47]. A top–down approach complementing bottom–up involvement and commitment and long-term coordinated efforts are necessary for reaching more sustainable changes.

Although our assessment of ten-year trends in antibiotic use has important policy implications, the study has limitations that need to be mentioned. The aggregate-level data used in this study provide no information about the length of treatment and the indication the drugs were used for, nor other specific patient-related data. The used quality indicators are intended for the outpatient setting, and despite efforts to exclude medicines solely available in an inpatient setting, there is a chance that, due to the nature of our data, there was a slight overestimation. Even though the use of DDD is a standardized measure, as with many calculations of an average, the DDD does not necessarily reflect the actual daily dosage. To surpass some of the limitations of the study, a ten-year period and several relative measures and their change over time were used to describe changes in patterns of antibiotic utilization. Therefore, even with several limitation of the study, the methodology adopted is widely used in similar studies and has been considered adequate to evaluate the antibiotic utilization trends.

## 4. Materials and Methods

Information about consumption of medicines were retrieved from the annual reports available on the Agency for Drugs and Medical Devices of the Republic of Serbia for a ten-year period between 2010 and 2019. Only medicines classified in the Anatomical Therapeutic Chemical (ATC) classification J01 group (antibacterials for systemic use) were included. The World Health Organization (WHO) ATC/DDD (Defined Daily Dose) methodology (Version 2020) was used to describe antibiotic utilization. The defined daily dose (DDD) is defined as “the assumed average maintenance dose per day for a drug used for its main indication in adults” [10]. The results were expressed as DDD per 1000 inhabitants per day (DID). All calculations were performed using the DDD values for the 2020 ATC/DDD version for each year of the study, to account for the DDD change [11] for several antibiotics that happened between 2005 and 2020 [48].

Trends in antibiotic use were also analyzed based on the route of administration (oral or parenteral) and AWaRe classification categories. The AWaRe classification describes three categories—Access, Watch and Reserve. The Access category includes 48 antibiotics recommended as the first- and second-line drugs for various infections, which have lower potential for invoking AMR. Drugs classified in the Watch (110) and Reserve (22 antibiotics) groups should be the focus for stewardship programs [20]. Antibiotics not listed in the WHO AWaRe classification were designated as “Unclassified”. The drug utilization 90% method was also used to rank antibiotics by volume of DIDs and specify the antibiotics accounting for 90% of the overall consumption at the beginning (2010), middle (2015) and the end of the study period (2019) [49].

Several quality indicators developed by the European Surveillance of Antimicrobial Consumption project [50] were determined—consumption of penicillins (J01C), cephalosporins (J01D), macrolides, lincosamides and streptogramins (J01F) and quinolones (J01M), expressed in DID. Consumption of beta-lactamase-sensitive penicillins (J01CE), combination of penicillins, including beta-lactamase inhibitor (J01CR), third- and fourth-generation cephalosporins (J01(DD + DE)) and fluoroquinolones (J01MA), expressed as the percentage of the total consumption of antibacterials for systemic use (J01), was also determined. The ratio of the consumption of broad-spectrum (J01(CR + DC + DD + (F-FA01))) to the consumption of narrow-spectrum penicillins, cephalosporins and macrolides (J01(CE + DB + FA01)) was calculated. The quality indicators are intended for the outpatient setting. In Serbia, 80% of all antibiotics in Serbia are prescribed in outpatient conditions [4]. The data used for this study contain wholesale data for the in- and out-patient settings. Therefore, in order to avoid an overestimation of figures when using wholesale data for reporting for the community sector, parenteral antibiotics that are only available for in-patient use according to the legislation by the Serbian agency for drug and medical devices (all parenteral antibiotics except J01GB03, J01GB06, J01CR02, J01CA01, J01DD04, J01DB04, J01DD02, J01DC02, J01FF01, J01FA10, J01FF01 and J01CE30) were excluded in the calculation of the abovementioned quality indicators. 

The statistical analysis was performed using the SPSS version 21.0 (IBM, Chicago, IL, USA) package. Results were presented as absolute values and percentages. Each of the antibiotic utilization series was explored independently for a trend over time by linear regression. All reported probability values were two-tailed and a 0.05 level of significance was considered to be appropriate. 

## 5. Conclusions

In Serbia, the total antibiotic utilization was found to be well above the European average. Several specific problem areas were identified, which requires further efforts to improve antibiotic prescribing. The present study provides information needed to facilitate antibiotic stewardship in Serbia further and stimulate policy makers to develop action plans, through identifying several problem areas that need to be addressed in order to optimize antibiotic use and tackle antimicrobial resistance.

## Figures and Tables

**Table 1:** Trends in the utilization of penicillins (J01C), cephalosporins (J01D), macrolides, lincosamides and streptogramins (J01F) and quinolones (J01M), expressed in DID.

Antibiotic Class	Antibiotic Utilization (DID) per Year										Regression	
	2010	2011	2012	2013	2014	2015	2016	2017	2018	2019	β	*p*
J01C	5.68	7.70	7.97	7.58	9.41	11.47	8.63	6.70	8.29	11.26	0.532	0.113
J01D	3.43	3.31	4.26	3.61	3.84	4.60	4.81	3.66	2.95	4.38	0.651	0.534
J01F	3.23	5.12	4.01	4.06	3.93	5.82	5.04	3.67	5.16	5.54	0.505	0.137
J01M	2.10	2.66	3.68	3.04	3.29	3.82	3.43	3.70	3.86	3.23	0.667	0.035

**Table 2:** Changes in the different quality indicators describing the relative use of beta-lactamase-sensitive penicillins (J01CE), penicillins, including beta lactamase inhibitor (J01CR), third- and fourth-generation cephalosporins (J01DD + J01DE) and fluoroquinolones (J01MA), as well as the ratio of utilization of broad-spectrum to narrow-spectrum penicillins, cephalosporins and macrolides (J01_B/N) of over 10-year period in Serbia.

Quality Indicator	Year										Regression	
	2010	2011	2012	2013	2014	2015	2016	2017	2018	2019	β	*p*
J01CE_% *	1.08%	0.00%	0.72%	0.47%	0.94%	0.41%	0.39%	0.39%	0.33%	0.26%	−0.57	0.086
J01CR_% *	6.57%	11.40%	7.41%	7.63%	7.83%	10.57%	10.15%	9.56%	18.87%	21.41%	0.772	0.009
J01DD + J01DE_% *	4.01%	2.55%	2.06%	2.09%	1.03%	2.83%	3.71%	5.12%	6.05%	5.53%	0.664	0.036
J01MA_% *	6.64%	7.49%	11.35%	8.94%	10.17%	10.11%	11.05%	14.79%	14.30%	11.16%	0.786	0.007
J01_B/N	1.42	2.34	1.37	1.92	1.15	2.07	1.95	2.39	6.00	4.16	0.692	0.027

* Expressed as percentage of the total consumption of antibacterials for systemic use (J01).

**Table 3:** Changes in utilization of individual agents for which a significant decreasing or increasing trend was found between 2010 and 2019.

ATC/DDD	2010	2019	% Change	β	Significance.
J01AA07-tetracycline	0.12	0.02	−82.42%	−0.85	0.002
J01AA12-tigecycline	0.001	0.01	510.00%	0.722	0.018
J01CR02-amoxicillin, clavulanic acid	1.09	5.93	442.00%	0.812	0.014
J01DB04-cefazolin	0.02	0.09	331.50%	0.643	0.045
J01DB05-cefadroxil	0.04	0.10	161.63%	0.728	0.064
J01DC04-cefaclor	0.13	0.02	−83.66%	−0.776	0.008
J01DD02-ceftazidime	0.02	0.04	92.65%	0.639	0.047
J01DD08-cefixime	0.65	1.40	114.55%	0.772	0.009
J01DD13-cefpodoxime	0.04 *	0.14	3296.25%	0.847	0.008
J01FA06-roxithromycin	0.5	0.12	−76.71%	−0.662	0.037
J01FA09-clarithromycin	0.94	1.64	74.79%	0.814	0.004
J01FF01-clindamycin	0.04	0.608	1421.00%	0.888	0.001
J01GB03-gentamicin	0.5	0.39	−21.58%	−0.695	0.026
J01MA02-ciprofloxacin	0.62	1.62	160.56%	0.689	0.028
J01MA12-levofloxacin	0.09 *	1.33	1375.56% *	0.919	*p* < 0.001
J01MB04-pipemidic acid	0.99	0.13	−86.26%	−0.884	0.001
J01XA01-vancomycin	0.03	0.051	70.00%	0.717	0.020
J01XX01-fosfomycin	0.02	0.095	373.20%	0.931	*p* < 0.001
J01XX08-linezolid	0.0004 *	0.006	1318.65%	0.98	*p* < 0.001

* Calculated from the first available year (2011).

**Table 4:** Drug utilization 90% (DU90%) profiles of the J01 group in the year 2010, 2015 and 2019 in Serbia (A—Access; Wa—Watch; NC—not classified).

No.	2010			2015			2019		
	ATC INN	DID	%	ATC INN	DID	%	ATC INN	DID	%
1	J01CA04-amoxicillin (A)	3.09	17.93	J01CA04-amoxicillin (A)	7.68	25.89	J01CR02-amoxicillin; clavulanic acid (A)	5.93	20.70
2	J01DB01-cefalexin (A)	2.40	13.91	J01DB01-cefalexin (A)	3.60	12.14	J01CA04-amoxicillin (A)	4.96	17.30
3	J01AA02-doxycycline (A)	1.38	8.00	J01FA10-azithromycin (Wa)	3.19	10.76	J01FA10-azithromycin (Wa)	2.83	9.87
4	J01CA01-ampicillin (A)	1.31	7.59	J01CR02-amoxicillin; clavulanic acid (A)	3.06	10.31	J01DB01-cefalexin (A)	2.29	7.99
5	J01FA10-azithromycin (Wa)	1.27	7.36	J01AA02-doxycycline (A)	2.20	7.42	J01FA09-clarithromycin (Wa)	1.64	5.74
6	J01CR02- amoxicillin, clavulanic acid (A)	1.09	6.34	J01MA02-ciprofloxacin (Wa)	1.90	6.42	J01MA02-ciprofloxacin (Wa)	1.62	5.64
7	J01MB04-pipemidic acid (NC)	0.99	5.74	J01FA09-clarithromycin (Wa)	1.46	4.92	J01AA02-doxycycline (A)	1.47	5.12
8	J01FA09-clarithromycin (Wa)	0.94	5.45	J01MB04-pipemidic acid (NC)	0.90	3.03	J01DD08-cefixime (Wa)	1.39	4.87
9	J01DD08-cefixime (Wa)	0.65	3.77	J01DD08-cefixime (Wa)	0.77	2.60	J01MA12-levofloxacin (Wa)	1.33	4.64
10	J01MA02-ciprofloxacin (Wa)	0.62	3.59	J01MA12-levofloxacin (Wa)	0.74	2.49	J01EE01-sulfamethoxazole; trimethoprim (A)	0.89	3.10
11	J01GB03-gentamicin (A)	0.50	2.90	J01CA01-ampicillin (A)	0.61	2.06	J01FF01-clindamycin (A)	0.61	2.12
12	J01FA06-roxithromycin (Wa)	0.50	2.90	J01GB03-gentamicin (A)	0.61	2.06	J01DD04-ceftriaxone (Wa)	0.47	1.65
13	J01DD04-ceftriaxone (Wa)	0.40	2.32				J01DC02-cefuroxime (Wa)	0.41	1.45
14	J01FA01-erythromycin (Wa)	0.35	2.03						
15	J01MA06-norfloxacin (Wa)	0.25	1.45						
DU90%	1–15	15.75	91.29	1–12	26.72	90.09	1–13	25.83	90.18
	16–43	1.50	8.71	13–48	2.94	9.91	14–49	2.81	9.82
Total	43	17.25	100.00	48	29.66	100.00	49	28.65	100.00

## Data Availability

The data presented in this study are openly available in Zenodo, at https://doi.org/10.5281/zenodo.4663719.
